# Tuberculoma of spine mimicking intramedullary tumour: A case report

**DOI:** 10.1016/j.ijscr.2020.09.097

**Published:** 2020-09-24

**Authors:** Karya Triko Biakto, Jainal Arifin, Gerald Wonggokusuma, Carlo Micelli

**Affiliations:** a Department of Orthopaedics and Traumatology, Faculty of Medicine of Hasanuddin University, Makassar, Indonesia; b Resident of Department of Orthopaedics and Traumatology, Faculty of Medicine of Hasanuddin University, Makassar, Indonesia

**Keywords:** Tuberculoma, Mimicking, Spinal cord tumour, Case report

## Abstract

**Introduction:**

Spinal intramedullary tuberculoma (IMTB) is a rare disease that accounts for 1 to 2/100,000 patients with tuberculosis [1]. This case demonstrated that this patient had atypical presentations compared to common tuberculosis considering that IMTB cases are really rare.

**Case presentation:**

A 19-year-old male presenting with back pain and weakness of both lower limbs, which started in the left lower limb and subsequently involved the right lower limb for three months. He had sensory impairment below T6 and hypoesthesia. He complained of urine hesitancy and a feeling of incomplete voiding of urine. He was on anti-tubercular treatment (ATT) for last two months. MRI exhibited hypointense portion on T1-weighted images. T2-weighted image also showed hypointensity at T6, which suggested for intramedullary mass. Excisional biopsy was suggestive for tuberculoma.

**Discussion:**

In TB-endemic country like in Indonesia, tuberculoma should be considered as a differential diagnosis for intramedullary SOL irrespective of age or presence of extra-cranial focus of TB. MRI is the optimal tool for evaluating and diagnosing IMTB at an early stage and useful in follow-up. However, IMTB imaging characteristics may vary with immune response of individual or the stage of the tuberculoma formation [3]. The aim of early surgery is to decompress the spinal cord and achieve improvement in neurological function.

**Conclusion:**

This case was reported to emphasize that early surgical decompression is required as a delay might cause damage to the spinal cord. This case also provides some evidences based data, thus contributing to the future research studies and clinical practice.

## Highlights

• Spinal intramedullary tuberculoma is a rare case.
• Clinical presentation of spinal IMTB is non-distinctive from other intramedullary lesions in the absence of systemic TB.
• Tuberculoma should be considered in the differential diagnosis of all intramedullary space-occupying lesion (SOL) in TB endemic countries.
• Early surgical decompression in selected cases may provide good long-term outcomes.

## 1 Introduction

Spinal intramedullary tuberculoma (IMTB) is a rare disease that accounts for 1 to 2/100,000 patients with tuberculosis [1]. Spinal tuberculoma is also an extrapulmonary manifestation of tuberculosis involving the central nervous system, and are characterized as extradural, intradural extramedullary, or intramedullary according to their location [2]. In the absence of systemic tuberculosis, clinical presentation is non-distinctive from other intramedullary lesions [6]. This case demonstrated that it was difficult to diagnose by the clinical presentation from other intramedullary lesions. The presentations were also unusual compared to common tuberculosis cases considering that intramedullary tuberculoma cases are really rare. This case has been reported in line with the SCARE criteria [7].

## 2 Case presentation

A 19-year-old male came to the hospital presented with back pain and weakness of both lower limbs of three months duration. It initially started in the left lower limb and subsequently involved the right lower limb. The patient also complained of urine hesitancy and a feeling of incomplete voiding of urine, but normal defecation. He came from primary health facility with acid-fast bacilli (AFB) smear-positive sputum. He was on anti-tubercular treatment for last two months with non-reactive HIV status, but there was no significant past medical history such as cough, night sweat, or decreased body weight. He denied any recent exposure from people who had tuberculosis.

**Fig. 1:**
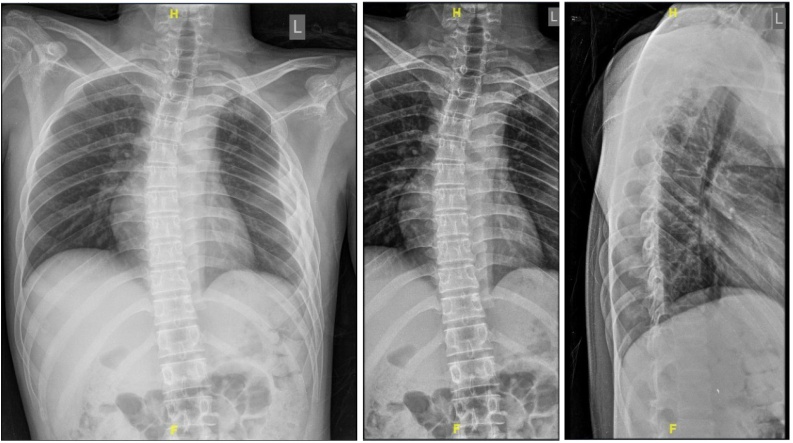
Plain X-Ray. Left: Chest X-Ray AP view showed homogenous consolidation at the right upper lung. Middle: Thoracolumbal X-Ray AP view showed normal finding. Right: Thoracic X-Ray lateral view demonstrated no abnormalities.

**Fig. 2:**
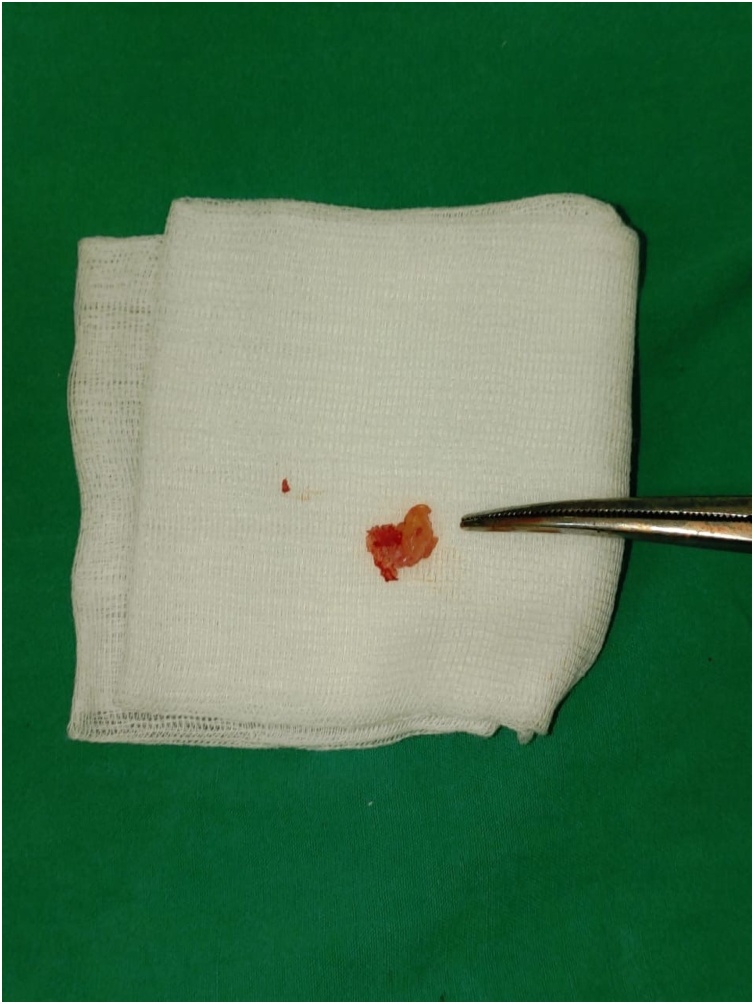
Resected specimen of intramedullary mass.

On examination, his mental functions and cranial nerve were normal. All of his reflexes are brisk and had a Babinsky sign for both of the lower limbs. Both of the upper limbs power were 5/5, whereas both of the lower limbs were 2/5. He had sensory impairment below T6 corresponding to vertebrae level and altered sensation with hypoesthesia. Laboratory findings revealed negative sputum examination for tuberculosis. Plain chest X-Ray, thoracolumbal, and lateral radiographs revealed normal finding (Fig. 1). Magnetic Resonance Imaging (MRI) on T1-weighted image exhibited hypointense portion, which appeared to surround the intermediate intensity lesion. The hypointense part represented the inflammatory reaction that occured around the lesion, whereas the isointensity in the middle reflected the presence of necrosis. Axial T1- weighted image showed an intradural hypointensity at T6 level (Fig. 5). T2-weighted image also showed low signal intensity at the vertebral level of thoracal VI, VII, and VIII. The hypointense portion reflected necrosis and also hypocellularity with increased macrophages and gliosis. The pathological layers were indistinguishable on T2‐weighted image (Fig. 2). The patient suspected with intramedullary mass spinal tumour. With the above imaging characteristics of intramedullary lesion, differential diagnosis of tuberculous granuloma, ependymoma and glioma were suggested.

**Fig. 3:**
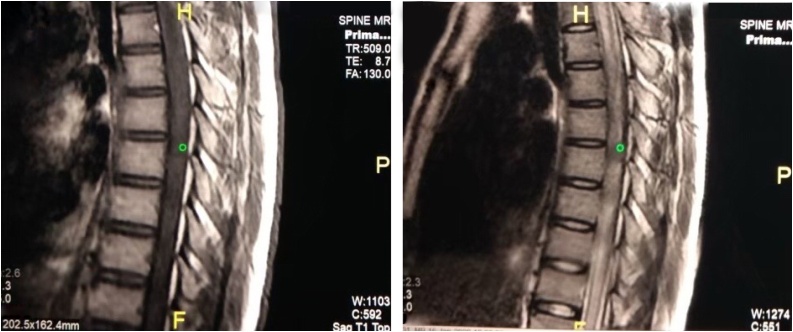
MRI sagittal view. Left: T1-weighted image showed intramedullary hypointense mass within T6. Right: T2-weighted image demonstrated low signal intensity in T6 level.

**Fig. 4:**
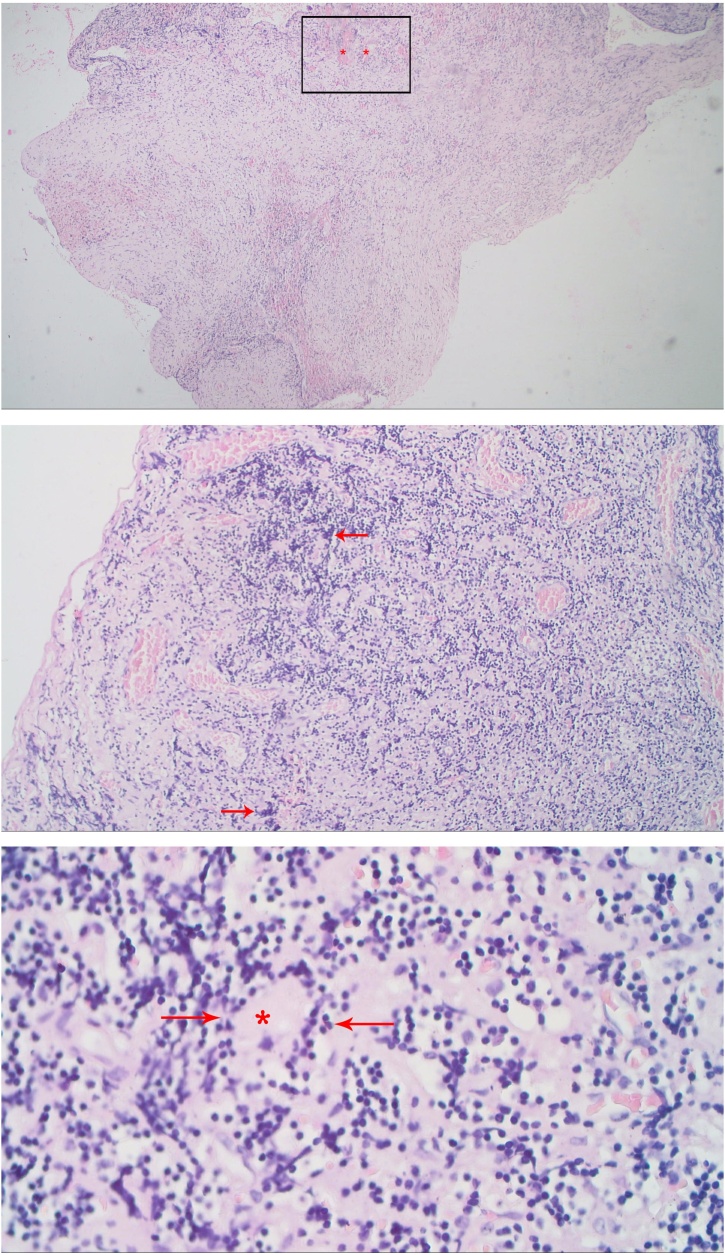
Histopathology. Top: This picture demonstrated granuloma with caseous necrosis. Histopathology revealed a granulomatous lesion with a central area of caseation in keeping with a tuberculoma (asterisk). Middle: Multiple giant cells and inflammatory cells at 10x magnification. Some histiocytes are forming multinucleated giant cells (arrow). Bottom: Epithelioid cells granuloma at 40x magnification. Edge of a necrotizing granuloma seen in a peripheral rim of epithelioid histiocytes (arrows) surrounding the central necrotic region (asterisk). External to the rim of histiocytes is an outer rim of lymphocytes and plasma cells.

Surgical resection was performed through posterior approach from T6 to T8 Level. Midline durotomy was perfomed and showed a thick mass intramedullary tumour at the vertebral level of thoracal VI, VII, and VIII (Fig. 3). The patient underwent posterior decompression of spinal cord and vertebral laminectomy with open biopsy. The tuberculoma was a brown greyish, multilobulated, irregular, but well-circumscribed mass. Excisional biopsy resulted as granulomatous chronic inflammation process, which was seen by the formation of epitheloid histiocytes (multinucleated langerhans type giant cells), with peripheral lymphocytes and plasma cells, and a central area of caseous necrosis. These pictures below were suggestive for tuberculoma of the spinal cord (Fig. 4).

**Fig. 5:**
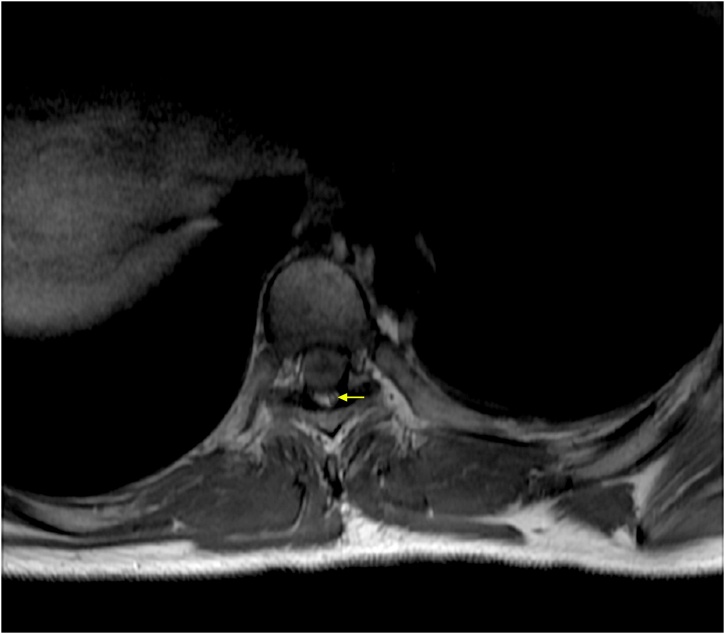
Axial T1-weighted MRI of T6 level, showing an intradural hypointense mass.

The patient’s lower limbs power gradually improved throughout one month after the surgery. Clinically, there was an improvement in neurological deficits and bladder dysfunction, but he still had mild hesitancy while passing urine. The patient was able to ambulate independently and resume productive activities.

## 3 Discussion

The thoracic segment is the most common site of IMTB and hematogenous spread is usually the main etiology. Most cases of intramedullary tuberculomas are subacute and present with progressive symptoms suggestive of a compressive myelopathy [8]. IMTB presents with symptoms of sensory loss, muscle weakness, and autonomic function loss depending on the spinal level involved [1,[9], [10], [11]]. *MacDonnell et al.* reported lower extremity weakness, back pain, bowel and bladder dysfunction, and paraesthesia as clinical presentations, which resemble to the presentations of this case [12]. The patient's motor function of both of the lower limbs decreased. He also had sensory impairment below T6 level. IMTB has been described in young immunocompromised as well as immunocompetent individuals due to HIV infection or immunosuppressive therapy. This patient was negative for both [13,14].

MRI is the optimal tool for evaluating and diagnosing IMTB at an early stage and useful in follow-up. However, MRI is sometimes non-specific and differentiation from malignant lesions is difficult, especially in the absence of TB in other body parts such as the lungs or lymph nodes. IMTB imaging characteristics may vary with immune response of individual or the stage of the tuberculoma formation. It means typical MRI signal characteristics may not be seen in all individuals [3]. *Chang et al.* and *Gupta et al.* showed that typical MRI characteristics of IMTB are hypo or isointense to cord in T1-weighted sequence with only an indirect sign of focal cord expansion and heterogenous intensity on T2-weigted sequence with central hypointensity and peripheral hyperintensity, which is described as target sign [15,16]. Meanwhile, this patient had atypical presentations that may lead to possible differential diagnosis such as ependymoma, glioma, or abscess caused by fungal organisms [17].

Tuberculoma should be considered in the differential diagnosis of all intramedullary space-occupying lesion (SOL) irrespective of age or presence of extra-cranial focus of tuberculosis in countries endemic to tuberculosis. These patients have been reported to respond well to anti-tuberculosis drug therapy with good functional recovery and most of the reported patients underwent surgery. The aim of the surgery is to decompress the spinal cord and to achieve improvement in neurological function. However, timely surgical decompression in selected cases provide good long-term outcomes and delayed surgery might be associated with worse outcomes. Mental or focal neurological changes during the follow-up must be examined [4,5].

## 4 Conclusion

In TB-endemic country like in Indonesia, tuberculoma should be considered as a differential diagnosis for intramedullary SOL regardless of age or evidence of systemic TB. The best treatment of intramedullary tuberculoma is still a topic of debate. Both surgical and medical treatments have given good results in different case. This case was reported to emphasize that early surgical decompression is required as a delay might cause irreversible damage to the spinal cord, leaving permanent neurological sequelae. This case could provide some evidence based data, thus contributing to the future research studies and clinical practice.

## Declaration of Competing Interest

There are no conflicts of interest.

## Funding

This study was funded independently.

## Ethical approval

This study was approved by the ethical board of Hasanuddin University of Makassar. Our patients has signed terms of consent to participate in the research of this original article. The institutional ethical committee has approved the publication of this original article.

## Consent

Written informed consent was obtained from the patient for publication of this case report and accompanying images. A copy of the written consent is available for review by the Editor-in-Chief of this journal on request.

## Author contribution

**Karya Triko Biakto:** Conceptualization, Methodology, Supervision, Data curation, Formal analysis, Validation, Resources, Funding acquisition, Project administration. **Jainal Arifin:** Conceptualization, Supervision, Formal analysis, Validation. **Gerald Wonggokusuma:** Conceptualization, Supervision, Formal analysis, Supervision, Validation, Writing - Original Draft, Writing - Review & Editing, Visualization. **Carlo Micelli:** Conceptualization, Software, Data curation, Formal analysis, Investigation, Validation, Writing - Original Draft, Writing - Review & Editing, Visualization.

## Registration of research studies

No.

## Guarantor

Gerald Wonggokusuma

## Provenance and peer review

Not commissioned, externally peer-reviewed
